# Mechanisms and Fitness Costs of Resistance to Antimicrobial Peptides LL-37, CNY100HL and Wheat Germ Histones

**DOI:** 10.1371/journal.pone.0068875

**Published:** 2013-07-23

**Authors:** Hava Lofton, Maria Pränting, Elisabeth Thulin, Dan I. Andersson

**Affiliations:** 1 Department of Medical Biochemistry and Microbiology, Uppsala University, Uppsala, Sweden; 2 Department of Cell and Molecular Biology, Uppsala University, Uppsala, Sweden; University of Massachusetts Medical School, United States of America

## Abstract

Antimicrobial peptides (AMPs) represent a potential new class of antimicrobial drugs with potent and broad-spectrum activities. However, knowledge about the mechanisms and rates of resistance development to AMPs and the resulting effects on fitness and cross-resistance is limited. We isolated antimicrobial peptide (AMP) resistant *Salmonella typhimurium* LT2 mutants by serially passaging several independent bacterial lineages in progressively increasing concentrations of LL-37, CNY100HL and Wheat Germ Histones. Significant AMP resistance developed in 15/18 independent bacterial lineages. Resistance mutations were identified by whole genome sequencing in two-component signal transduction systems (*pmrB* and *phoP*) as well as in the LPS core biosynthesis pathway (*waaY,* also designated *rfaY*). In most cases, resistance was associated with a reduced fitness, observed as a decreased growth rate, which was dependent on growth conditions and mutation type. Importantly, mutations in *waaY* decreased bacterial susceptibility to all tested AMPs and the mutant outcompeted the wild type parental strain at AMP concentrations below the MIC for the wild type. Our data suggests that resistance to antimicrobial peptides can develop rapidly through mechanisms that confer cross-resistance to several AMPs. Importantly, AMP-resistant mutants can have a competitive advantage over the wild type strain at AMP concentrations similar to those found near human epithelial cells. These results suggest that resistant mutants could both be selected *de novo* and maintained by exposure to our own natural repertoire of defence molecules.

## Introduction

Antibiotic resistance in pathogenic bacteria is spreading at an alarming rate and we are in urgent need of new antimicrobial drugs. Interesting new candidates for future antimicrobial drugs are antimicrobial peptides (AMPs) and since their discovery in the cecropia larvae in 1980 [1], there has been major interest in developing antimicrobial drugs from peptide scaffolds. These small molecules are part of the innate immune system and found in abundance in nature, in organisms ranging from bacteria to humans. AMPs protect the producing organisms by directly killing invading pathogens or by functioning as signalling molecules and immune system modulators. They are therefore often referred to as host defence peptides and are often broad-spectrum and potent in their antimicrobial activities [2]. In addition, it has been suggested that resistance development is unlikely given that AMPs often act non-specifically on conserved targets like the bacterial membrane, resulting in a high cost of resistance [3].

However, little is in fact known about bacterial ability to develop resistance to AMPs and what would happen if we were to begin using molecules of our own innate immune system as drugs against microbial infections. As previously discussed and modelled, high usage of AMPs might, as for antibiotics, lead to selection and spread of resistant pathogens [4]. In addition, a few recent studies describe resistance development to AMPs in bacteria, demonstrating that resistance development is possible. Perhaps the most important example comes from Perron *et al* [5], who serially passaged several lineages of *Pseudomonas fluorescens* and *Escherichia coli* in progressively increasing concentrations of the peptide Pexiganan, a well-characterized AMP developed for use as a pharmaceutical [6], [7]. Most of the 24 bacterial lineages developed significant resistance against Pexiganan over the course of the experiment, a 32–512 fold and 2–64 fold increase in resistance level compared to the parental strains for *P. fluorescens* and *E. coli*, respectively [5]. More recently, a study by Habets *et al* [8], using *Staphylococcus aureus* demonstrated (again using Pexiganan and a similar serial passage method as above) that *S. aureus* was able to develop resistance at a fitness cost, which was then easily compensated for with further cycling. This same study showed that cross-resistance to the human-neutrophil-defensin-1 AMP developed. Unfortunately, in both examples the mutants were not characterized in detail and the resistance mutations never identified. Additionally, two peptides already in use, nisin and colistin, have reports of resistance emerging [9], [10]. It also is becoming increasingly clear that slow growing variant forms of bacteria (so called small colony variants) are important during several disease conditions such as cystic fibrosis and device related infections [11], [12]. In many cases, these small colony variants have been demonstrated to better withstand several AMPs and antibiotics, and may also be selected by such molecules in the body [12]. Thus, a serious concern is that by developing AMPs for widespread therapeutic use we might select for bacteria that can better withstand our own immune defence. This could ultimately render us more susceptible to infection by these bacteria.

By studying resistance development to AMPs we hope to gain insight into the mechanisms and general physiological effects on the bacteria that could hopefully enable the development of efficient antimicrobial drugs. In this study, we have performed an evolution experiment in which *S. typhimurium* LT2 was subjected to successively increasing concentrations of three different AMPs: LL-37, CNY100HL and Wheat Germ Histones. LL-37 is a human AMP belonging to the cathelicidin polypeptide family [13]. It is one of the most well studied AMPs and is known to be active in the innate immune system [14], [15]. CNY100HL is a modified AMP derived from the C3 complement peptide CNY21 [16]. These two peptides are thought to perturb the lipid bilayer of the bacterial membrane, ultimately forming pores and disrupting the membrane. The histones are extracted from wheat germ, and consist of a mixture of different histones and shorter histone peptides. Histones are not typically on the list of AMPs since they are normally busy structuring DNA strands, but several recent studies have demonstrated that histones or histone derived peptides have an important role in antimicrobial defence [17].

In this study, resistant mutant clones were isolated and the resistance mutations identified by whole genome sequencing. The original mutants as well as reconstituted resistant mutants were further characterized in terms of fitness, resistance level and cross-resistance. Over time, *S. typhimurium* evolved resistance to all tested AMPs, in most cases resulting in a concomitant fitness decrease. Mutations were identified in two-component signal transduction pathways as well as in the lipopolysaccharide biosynthesis pathway. Certain mutations (*e.g*. in *waaY*), conferred cross-resistance to all the peptides used in this study, implying that use of one peptide might confer collateral resistance to other classes of peptides.

## Materials and Methods

### Bacterial Strains, Media and Antimicrobial Peptides

All strains used in this study are derivatives of *Salmonella enterica* serovar Typhimurium LT2 (referred to as *Salmonella typhimurium*) and are listed in Table 1. When not specified, bacteria were grown in Luria Bertani broth (LB) and plated on Luria agar (LA, Sigma-Aldrich). When appropriate, the medium was supplemented with the following antibiotics: kanamycin (Kan, 50–100 mg/L), chloramphenicol (Chl, 30 mg/L) and tetracycline (Tet, 7.5–15 mg/L), all from Sigma-Aldrich. To prepare refined LB, LB without NaCl was subjected to ion exchange chromatography as described [18]. The medium was passed through a column of diameter 3.2 cm containing 30 mL of pre-charged and washed DEAE-Sephacel resin (Sigma-Aldrich, wet bead size 40–160 µM), using a peristaltic pump to maintain a flow rate of 1 mL/min. The refined LB was divided into ∼20 mL aliquots and stored in −20°C. Dilutions for viable counts were performed in phosphate buffered saline (PBS; 13 mM phosphate, 137 mM NaCl, pH 7.4). A mixture of wheat germ histones (WGH) in 0.1% acetic acid was kindly supplied by Lars-Olof Hedén at Lund University, Lund, Sweden (acid extracts of homogenized wheat germ, fractions 50–57, isolated as described [19]. CNY100HL (CKYILLLRRQLARAWRRGLR) [16], was kindly provided by Martin Malmsten (Uppsala University, Uppsala, Sweden) or purchased from Innovagen AB (Lund, Sweden), and LL-37 (LLGDFFRKSKEKIGKEFKRIVQRIKDFLRNLVPRTES) was synthesized by Innovagen AB (Lund, Sweden). These peptides (and all further stock solutions) were dissolved in distilled water.

**Table 1:** Strains and plasmids used in this study.

Strain	Genotype	Origin and description
DA6079	*Salmonella typhimurium* LT2, *cobI*344::Tn*10d*Chl	Lab collection
DA6192	*S. typhimurium* LT2	Lab collection, wild type
DA15110	*galK*::*yfp*-*bla*	22
DA15111	*galK*::*cfp*-*bla*	22
DA16874	*cobI*344::Tn*10d*Chl, *mutL*(delbp201–206, aa 68–69), *pmrB*(H164R), *waaY*(H152Y),>60 additional mutations	This work, original LL-37 resistant isolate 1, mutator
DA16875	*cobI*344::Tn*10d*Chl, *pmrB*(R13H), *waaY*(del bp17→FS) 3912438	This work, original CNY100HL resistant isolate 1
DA17610	*cobI*344::Tn*10d*Chl, *phoP*(D23N) *ffh*(L218Q)	This work, original CNY100HL resistant isolate 1
DA17847	*cobI*344::Tn*10d*Chl, *waaYZ*(del bp306–196 nt into waaZ) 3911421–3912154), *ytfN*(L1251F)	This work, original LL-37 resistant isolate 2
DA22427	*waaY*(del bp17→FS), *tdh*(scar)	This work, reconstituted single
DA22431	*tdh* (scar)	This work, reconstituted control
DA23040	*mutL*(delbp201–206, aa 68–69) (del bp 4600685–4600690), 4597041::Kan	This work, reconstituted single
DA23042	4597041::Kan	This work, reconstituted control
DA23175	*pmrB*(R13H), *adi*(scar)	This work, reconstituted single
DA23177	*adi*(scar)	This work, reconstituted control
DA23179	*waaY*(del bp17→FS), *pmrB*(R13H), *tdh*(scar), *adi*(scar)	This work, reconstituted double
DA23299	*tdh*(scar), *adi*(scar)	This work, reconstituted control
DA23301	*waaY*(del bp17→FS), *phoP*(D23N), *tdh*(scar), 1327454(scar)	This work, reconstituted double
DA23305	*tdh*(scar), 1327454(scar)	This work, reconstituted control
DA23307	*phoP*(D23N), 1327454(scar)	This work, reconstituted single
DA23309	1327454(scar)	This work, reconstituted control
DA23899	*waaY*(del bp17→FS), *pmrB*(R13H), *phoP*(D23N), *tdh*(scar), *adi*(scar), 1327454(scar)	This work, reconstituted triple
DA23902	*tdh*(scar), *adi*(scar), 1327454(scar)	This work, reconstituted control
DA24144	*adi*(scar), 1327454(scar)	This work, reconstituted control
DA24301	*pmrB*(R13H), *phoP*(D23N), *adi*(scar), 1327454(scar)	This work, reconstituted double
DA23410	*tdh* (scar), *galK*::*yfp*-*bla*	This work, for competition assay
DA23414	*adi*(scar), *galK*::*yfp*-*bla*	This work, for competition assay
DA23418	*tdh*(scar), *adi*(scar), *galK*::*yfp*-*bla*	This work, for competition assay
DA23423	*tdh*(scar), 1327454(scar), *galK*::*yfp*-*bla*	This work, for competition assay
DA23426	1327454(scar), *galK*::*yfp*-*bla*	This work, for competition assay
DA24148	*waaY* (del bp17→FS), *tdh* (scar), *galK*::*cfp*-*bla*	This work, for competition assay
DA24152	*pmrB*(R13H), *adi*(scar), *galK*::*cfp*-*bla*	This work, for competition assay
DA24156	*waaY* (del bp17→FS), *pmrB*(R13H), *tdh*(scar), *adi*(scar), *galK*::*cfp*-*bla*	This work, for competition assay
DA24161	*waaY* (del bp17→FS), *phoP*(D23N), *tdh*(scar), 1327454(scar), *galK*::*cfp*-*bla*	This work, for competition assay
DA24164	*phoP*(D23N), 1327454(scar), *galK*::*cfp*-*bla*	This work, for competition assay
DA24577	*waaY* (del bp17→FS), *pmrB*(R13H), *phoP*(D23N), *tdh*(scar), *adi*(scar), 1327454(scar), *galK*::*cfp*-*bla*	This work, for competition assay
DA24583	*pmrB*(R13H), *phoP*(D23N), *tdh*(scar), 1327454(scar), *galK*::*cfp*-*bla*	This work, for competition assay
DA24593	*tdh*(scar), *adi*(scar), 1327454(scar), *galK*::*yfp*-*bla*	This work, for competition assay
DA24603	*adi*(scar), 1327454(scar), *galK*::*yfp*-*bla*	This work, for competition assay
**Plasmid**		
pSIM5-Tet	Temperature-controlled λ-red system	Lab Collection

a All strains are derived from *S. typhimurium* LT2.

b Exact position of scar sequences can be found in Table S2.

c Only selected mutations confirmed by PCR shown here. For a full list of mutations, see Tables S3, S4, S5, and S6.

FS, frame shift; del, deletion.

### Antimicrobial Peptide Susceptibility Assays

#### Bacterial killing over time

Bacteria were grown overnight in 20 mM sodium phosphate buffer supplemented with 0.1% w/v Trypticase Soy broth (TSB; Becton, Dickinson and Company) and diluted in the same medium to approximately 2–4×10^6^ cfu/mL. Sodium phosphate buffer +0.1% TSB is referred to as NaPB from here on. 90 µL bacterial solution was then mixed with 10 µL peptide solution or water (positive control) in microcentrifuge tubes and incubated in a shaking incubator at 180 rpm and 37°C. Final concentrations of the different peptides ranged between 2–10 mg/L for CNY100HL, 5–25 mg/L for WGH and 5–25 mg/L for LL-37. Samples of 10 µL were removed from the tubes after 0, 1, 2, 4 and 24 hours to determine the number of viable cells. Survival at each time point is given as the average number of cfu/mL (±SEM) of at least two separate experiments.

#### Minimum inhibitory concentration (MIC)

MIC measurements were performed in refined LB or NaPB in 96-well plates (round bottom; Nunc A/S, Roskilde, Denmark) in a total volume of 100 µL or 50 µL. For all peptides a twofold dilution series was prepared in duplicate. Either 1 µL of a bacterial population or an isolated colony was inoculated in the assay medium and grown overnight. For refined LB, bacteria were subsequently diluted and grown to OD ∼0.2, diluted 100 times in refined LB and then 90/45 µL was added to each peptide well and to control wells without peptide. For experiments in NaPB, the overnight cultures were diluted 100 times and directly added to the peptide wells. The plates were incubated at 37°C in a shaking incubator at 180 rpm and the MIC was determined after approximately 16–20 h by visually examining the plate. The MIC was set to the lowest concentration of peptide resulting in no visible growth.

#### Optical density measurement in the presence of AMPs

Twofold dilution series of peptides in water (CNY100HL 40-0.31 mg/L; LL-37 50-0.39 mg/L; WGH 100-0.78 mg/L) were added in a total volume of 10 µL to wells in a bioscreen plate. 90 µL refined LB was added to each well. Bacteria were grown overnight in refined LB and 1 µL (approximately10^6^ cfu) was added to each well. Blank wells with only media and control wells without peptide were added in each experiment. OD_600_ values were measured every 4^th^ min for 8 h in a Bioscreen C analyzer (Oy Growth Curves Ab Ltd, Helsinki, Finland) at 37°C with shaking prior to each measurement.

### Serial Passage Experiment and Isolation of Mutants

The experimental evolution assay was carried out in 96-well plates with wild type *S. typhimurium* carrying a Chl-cassette in the *cob* operon (strain DA6079). 100 µl of refined LB supplemented with peptide was added to each of 6 wells per peptide. In addition, each plate contained 8 positive control wells (bacterial culture without peptide) and 4 negative controls (refined LB). The serial passage was started at a peptide concentration that reduced growth but did not kill the bacteria as determined by MIC measurements and OD-measurements during growth in the presence of peptide (Table S1 and Figure S1), corresponding to 12.5 mg/L for LL-37, 10 mg/L for CNY100HL and 20 mg/L for WGH. Each replicate well in the experiment was inoculated with bacteria from independent cultures started from less than 5000 cells and the plate was incubated with shaking at 37°C. Every day bacteria from each lineage were transferred to fresh medium in a new 96-well plate to allow approximately 6–7 doublings per growth cycle. After transfer, 10 µl DMSO was added to each culture well and the 96-well plates were saved in –80°C. If the bacteria in a well did not grow after transfer, the lineage was re-started from the most recent passage with growth. As a result, certain lineages were evolved for fewer generations than others. When a lineage had survived at least 10 subsequent transfers, the peptide concentration was increased by 50%. When a bacterial lineage survived significantly higher peptide concentrations than at the start of the experiment, decreased susceptibility was confirmed by MIC assays on 1 µL samples from the population. Clones from each lineage were isolated by streaking for single cells on LA-Chl and cultures from single cells were saved at –80°C. All subsequent experiments were performed on the single clones.

### DNA Isolation, Sequencing and PCR

Genomic DNA isolation was performed using the Genomic Tip 100G DNA kit (Qiagen) according to the manufacturer’s instructions. Four resistant clones from the cycling experiment were sent to BGI (Beijing Genome Institute, China) for whole-genome sequencing using Illumina sequencing technology. The CLC Genomics Workbench software (5.5.1, CLC bio, Aarhus, Denmark) was used to assemble sequencing reads and detect regions with sequence variation (quality based variant detection) and regions with no coverage as compared to *S. typhimurium* LT2 wild type strain (publically available at NCBI, NC_003197). The cutoff frequency was set to 75%. Phusion High-Fidelity DNA polymerase (Finnzymes) or TaqGold polymerase (Fermentas) were used in PCR reactions. Purification of PCR products was performed with the Fermentas GeneJet PCR DNA and Gel Band Purification Kit. DNA sequencing of PCR products was performed by Eurofins MWG operon (Ebersberg, Germany).

### Strain Construction

Selected mutations were reconstituted in wild type genetic background by standard genetic techniques (Table 1). In brief, a Kan resistance marker was introduced 3–10 kb away from the mutation of interest either by a temperature-controlled linear transformation system or P22 transduction of relevant Kan markers from our strain collection [20]. New P22-lysates were then prepared on the constructed strains and used to infect the wild type background. From each transduction an congenic strain pair was saved, carrying the mutation and the resistance marker or only the resistance marker. The Kan cassette was subsequently removed by introducing plasmid pcp20 expressing Flp recombinase that act on Flp recombination sites flanking the resistance cassette, leaving an 85-nt scar (Table S2) [20], . All reconstructed strains were confirmed by PCR and sequencing of the transferred region/gene of interest.

### Fitness Measurements

Bacteria were grown overnight in three different media, Mueller-Hinton (MH, Becton, Dickinson and Company), refined LB (the medium used in mutant selection) and NaPB. The overnight cultures were then diluted in the same medium to ∼1–3×10^6^ cfu/mL, and for each strain 300 µL were added in quadruplicate to a 96-well plate. Media blanks were added in each experiment to enable subtraction of the absorbance values of the medium. Growth of the samples was monitored at 37°C with shaking for 17 h with a Bioscreen C Analyzer (Oy Growth Curves Ab Ltd). OD_600_ measurements were taken every 4 min. Calculations are based on OD_600_ values between 0.02 and 0.2 wherever growth was observed to be exponential. The parental strain and selected resistant mutants were assayed in the same experiments and the experiments were repeated three separate times. Relative growth rates were calculated by dividing the generation time of the parental strain with the generation time of the mutants from the same experiment.

### Competition Assays

Independent overnight cultures (in NaPB) of bacteria genetically tagged with neutral *cfp* and *yfp* genes expressing fluorescent protein markers (Cfp/Yfp) [22], were grown and used for competition experiments. Congenic peptide resistant mutants (*cfp*) and their respective susceptible wild type controls (*yfp*) were mixed in approximately a 1∶1 ratio and diluted 1/100 in NaPB for a total volume of 100 µL (∼10^6^ bacteria of each strain). The mixed cultures were maintained in a 96-well plate and diluted 100-fold every 24 hours with fresh medium supplemented with different AMP-concentrations (between 0 and 10 mg/L) for 4–5 passages (roughly 30 generations). Between two and four separate competition experiments were performed in duplicates for each strain pair. The progress of the competition was monitored every 7th generation by taking 50 µL of sample and diluting it in 0.5 mL PBS sterilized through a 0.2 µm Filtropur filter (Sarstedt). The number of cells in each population was measured in a fluorescence activated cell sorter (BD FacsAria) that has the ability to count many more individual cells than standard competition methods. At each daily transfer, separate (non-competing) control cultures were started so that the pure fluorescent culture could be properly gated using the FacsAria software. The ratio of the resistant strain to the sensitive strain was plotted against generations of growth. A positive slope means the resistant strain is winning while a negative slope means the resistant strain is losing in the competition. The selection coefficients were deduced from the slope using the regression model as described [23], [24].

### Mutation Rate Determination

General mutation rates were determined for the parental strain and the original peptide resistant mutants as the mutation rate to rifampicin resistance [25]. Independent cultures were started of each bacterial strain by diluting a 24h-culture to approximately 10^3^ cells/mL in LB and aliquoting 300 µL bacterial solution in 10 to 15 independent 10 ml tubes. After 24 h incubation at 37°C with shaking (180 rpm), 200 µL of each of the 10–15 replicates was spread on LA plates supplemented with rifampicin (100 mg/L). If necessary, the 200 µL sample was diluted in PBS before plating. The viable count in 4–6 cultures was determined from a 50 µL sample. The number of colonies was counted after 24 h incubation at 37°C and the mutation rate was calculated using the median method of Lea-Coulson [26].

### Cross-resistance Studies

Cross-resistance to antibiotics for the different original and reconstituted mutants was measured by standard Etest strips (AB bioMérieux, Solna, Sweden), and compared to the susceptible parental strain/s. Bacteria were grown overnight in MH broth and diluted 100-fold before spreading evenly over MH agar plates. The Etest strip was placed on the agar and the results were analysed after ∼24 hours. Cross-resistance against the AMPs used in this study was measured by MIC assays, killing assays and competition assays as described above.

### New DNA Sequencing Data

All new sequence data has been deposited in GenBank with the following accession number: SRP023134.

## Results

### Isolation of Mutants

Serial passage of bacteria was carried out to select *S. typhimurium* mutants with reduced susceptibility to the AMPs LL-37, CNY100HL and WGHs. Several independent bacterial lineages were grown and passaged in refined LB in successively increasing concentrations of peptides. After approximately 400–500 generations of growth most bacterial lineages were able to grow in a peptide concentration several times higher than at the start of the experiment. MIC-assays in refined LB were then performed to confirm differences in AMP susceptibility between AMP-cycled lineages and lineages that had only been cycled in media without AMPs. Adaptation to the media seems to confer a small protection against peptides (see footnote Table 2), but the increase in resistance was always higher for the peptide-cycled lineages than for the control lineages. In subsequent experiments, mutants were compared to the parental wild type strain. Table 2 presents the number of generations of growth for each bacterial lineage, the AMP-concentrations in the wells at the start and end of the experiment, and the MICs of AMPs for selected lineages.

**Table 2:** AMP concentrations and AMP MICs for the different bacterial lineages before and after experimental evolution in refined LB.

Lineage	Start AMP-conc. (mg/L)	End AMP-conc. (mg/L)	No. of generations	MIC after cycling (mg/L)^a^
LL-37 1	12.5	63.3	511	75
LL-37 2	12.5	63.3	511	75– >75
LL-37 3	12.5	42.2	490	ND
LL-37 4	12.5	42.2	504	ND
LL-37 5	12.5	63.3	553	75–>75
LL-37 6	12.5	63.3	518	75
CNY100HL 1	10	33.8	476	10
CNY100HL 2	10	22.5	462	ND
CNY100HL 3	10	33.8	532	10–20
CNY100HL 4	10	33.8	469	10–30
CNY100HL 5	10	22.5	413	ND
CNY100HL 6	10	22.5	441	ND
WGH 1	20	200	567	>300
WGH 2	20	200	560	>300
WGH 3	20	200	553	>300
WGH 4	20	200	560	>300
WGH 5	20	200	553	>300
WGH 6	20	200	560	>300

a Peptide-MIC for positive control lineages after cycling (bacteria cycled without peptide): LL-37, PC 2 = 25–50 mg/L, PC 4 = 50 mg/L; CNY100HL, PC 6 = 5–10 mg/L; WGH, PC 2 = 6.25–12.5 mg/L, PC 7 = 12.5 mg/L.

ND, not determined.

MIC determinations were performed on a population of cells (compare Table 4).

After 490–550 generations, four out of six LL-37-treated lineages grew well in 63.3 mg/L, as compared to poor or no growth in 6.25–12.5 mg/L before the start of the experiment (Table 2, Figure S1). The MIC in refined LB of LL-37 for selected lineages was 75 to >75 mg/L, while the MIC before cycling was ∼25 mg/mL (Table 2, Table S1). After 410–530 generations three of the CNY100HL-treated lineages survived in 33.8 mg/L CNY100HL (Table 2). Before cycling, growth was strongly reduced at 10 mg/L (Figure S1). The MICs of CNY100HL in refined LB for the most resistant lineages was 10–30 mg/L, as compared to 5 mg/L for the wild type strain (Table 2, Table S1). Increased resistance to WGH occurred relatively fast, and already after 320 generations all lineages survived 100 mg/L, and after approximately 550 generations of growth all lineages grew in 200 mg/L WGH, a concentration 10 times higher than the initial concentration. The MIC of WGH in refined LB for all WGH-treated lineages was even higher, >300 mg/L, more than 20 times the MIC of WGH for the parental strain (*i.e.* both the original wild type strain and passaged controls, Table 2, Table S1). Individual clones were isolated from lineages that were able to survive high peptide concentrations. Four of these clones, two LL-37, one WGH and one CNY100HL resistant isolate were selected for further studies.

### Characterization of Mutants

#### Time-kill kinetics

**Figure 1:**
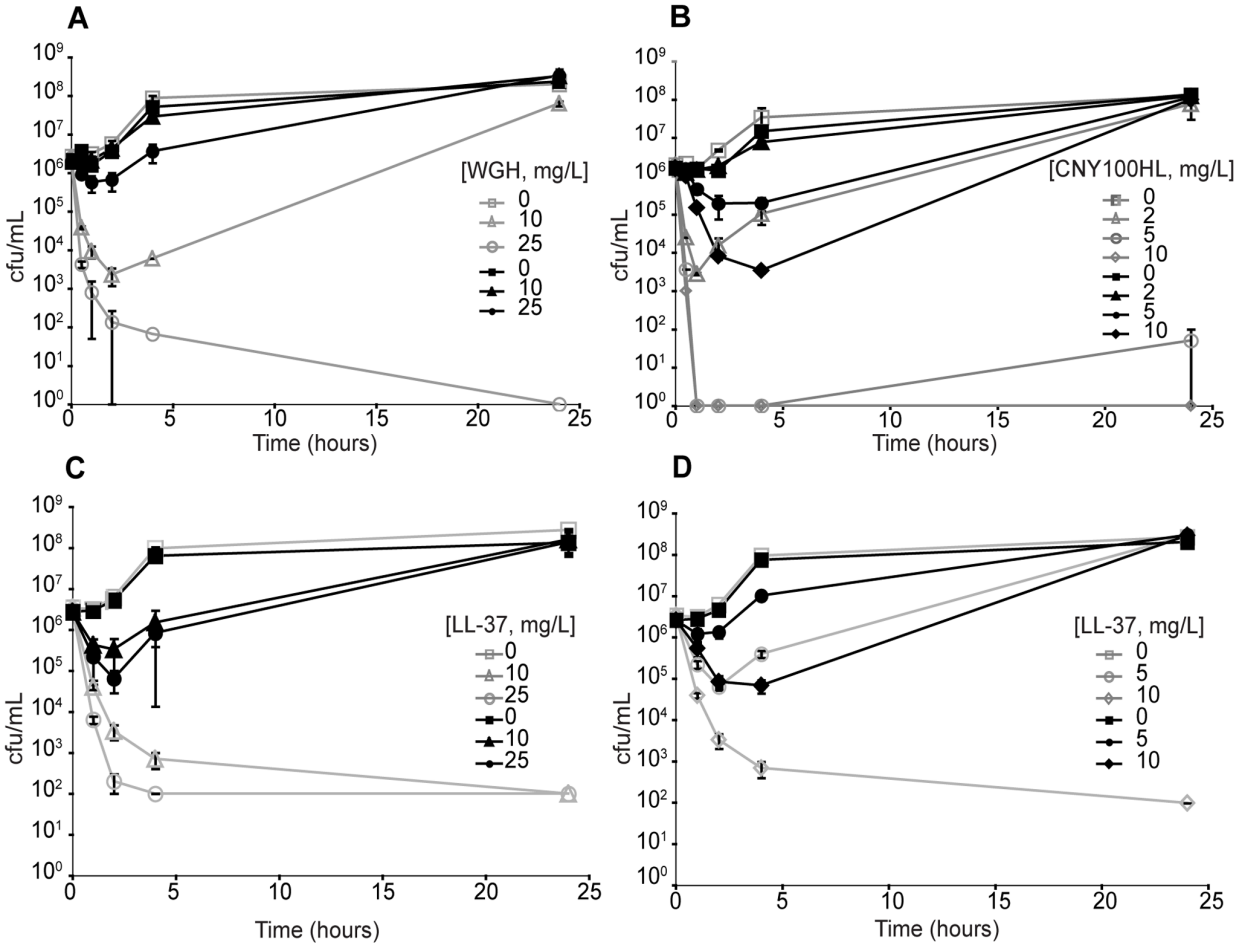
Susceptibility of the original mutants to the AMPs used in the original selection experiment. The curves represent the change in number of viable cells (log scale) over time at different concentrations of AMPs in NaPB. (A) WGH resistant isolate 1 (DA16875)+WGH; (B) CNY100HL resistant isolate 1 (DA17610)+CNY100HL; (C) LL-37 resistant isolate 1 (DA16874)+LL-37; (D) LL-37 resistant isolate 2 (DA17847)+LL-37. Open symbols, parental wild type strain (DA6079); filled symbols, original cycled resistant strains. The error bars represent the SEM (±).

Refined LB is time consuming to produce and yields are low. For large scale testing of the mutants we therefore used a sodium phosphate buffer similar to that used in radial diffusion assays for AMP activity [18], [27], only modified with 0.1% TSB to allow growth of the bacteria (designated NaPB). All mutants showed an increased resistance in both media, even though resistance levels were generally slightly lower in NaPB medium than refined LB. Increased peptide resistance of the originally isolated mutants was confirmed by examining time-kill kinetics in NaPB (Figure 1). The WGH resistant isolate DA16875 was barely affected at 25 mg/L WGH while this concentration efficiently killed the wild type control which was also more susceptible 10 mg/L WGH. The LL-37 resistant isolates DA17874 and DA16874 survived about 2–3 logs better after 4 hours and 6 logs better after 24 hours in 10 mg/L of LL-37 compared to the wild type control, and DA16874 also survived equally well in 25 mg/L LL-37. The CNY100HL resistant isolate DA17610 survived in 5 and 10 mg/L CNY100HL whereas the wild type was killed at both these concentrations.

#### Whole genome sequencing and genetic reconstitution of mutations

The genomes of the four selected peptide resistant isolates were sequenced using Illumina sequencing technology with a 60- to 90- times coverage. LL-37 resistant isolate 1 (DA16874) contained more than 80 different mutations, an indication of an abnormally high mutation rate, while the other three strains carried 2–3 mutations (, S4, S5, and S6). By comparing the different strains, three genes were selected as candidates for contributing to resistance in the different strains. Different mutations in *waaY* (and/or *waaZ)* were identified in three (DA16874, DA16875, DA17847) of the four resistant mutants (Tables S3, S4, S5), as well as in additional clones from other lineages (data not shown). The *waaY* (also designated *rfaY*) gene product modifies the LPS core region by heptose-phosphorylation [28], [29]. Mutations in *pmrB* were found in two strains (DA16874 and DA16875), while a mutation in *phoP* was one of only two mutations identified in the CNY100HL resistant isolate DA17610 (Table S6). Both *pmrB* and *phoP* are part of two component signal transduction systems regulating genes involved in modifying the bacterial membrane by *e.g.* masking phosphate groups or influencing membrane fluidity [30], [31].

**Figure 2:**
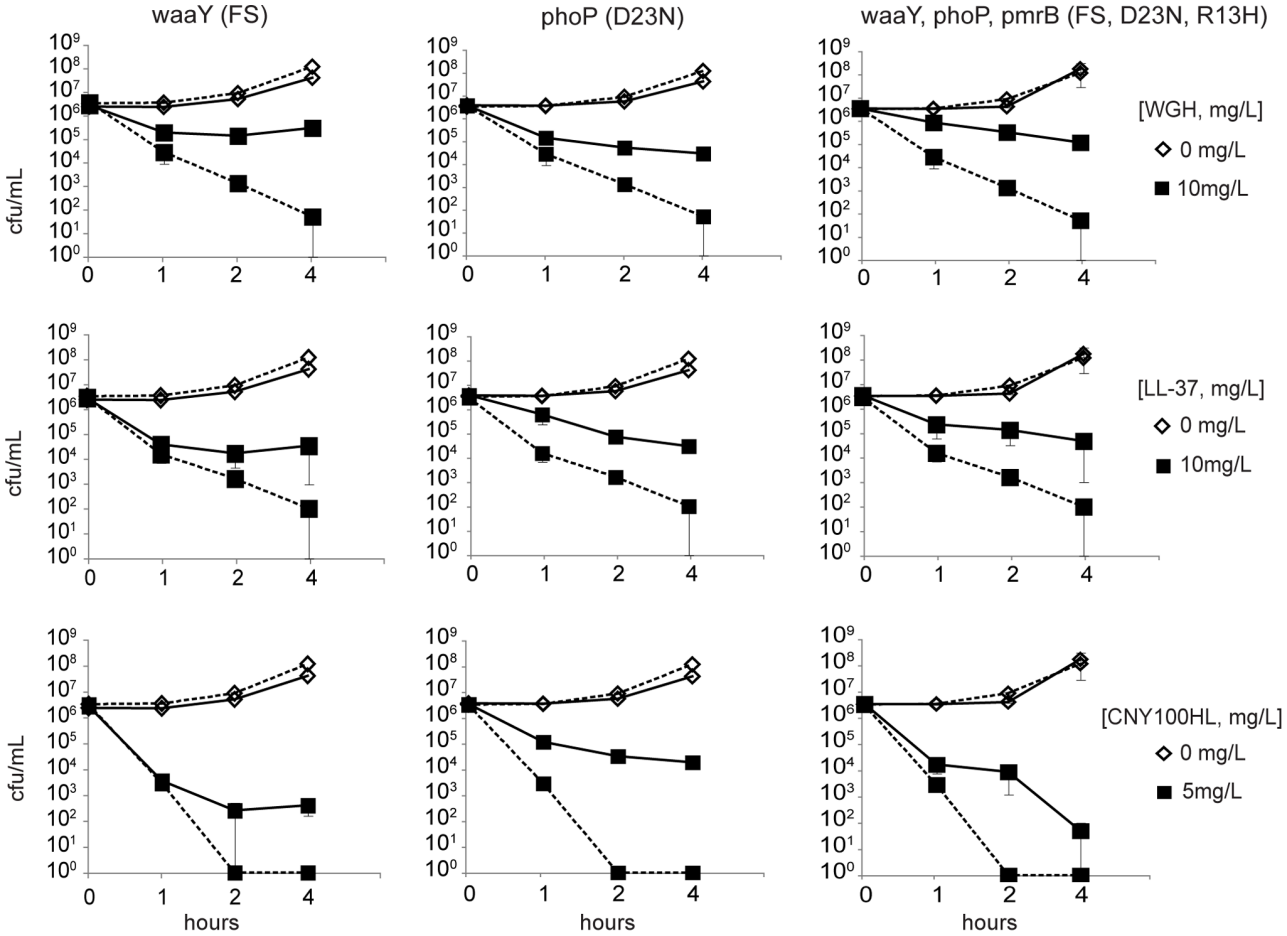
Susceptibility of reconstituted mutant strains to WGH, LL-37 and CNY100HL. The curves represent the change in number of viable cells (log scale) over time at different concentrations of AMP in NaPB. Left panel, *waaY* (FS) mutant DA22427; middle panel, *phoP* (D23N) mutant DA23307; right panel, *waaY*, *phoP*, *pmrB* (R13H) triple mutant DA23899 treated with from top to bottom: WGHs, LL-37 and CNY100HL. The susceptible wild type strain (DA6192) is included in all graphs. Dashed lines represent wild type and solid lines reconstituted mutants. The data is the average of at least 2 separate experiments ±SEM.

**Figure 3:**
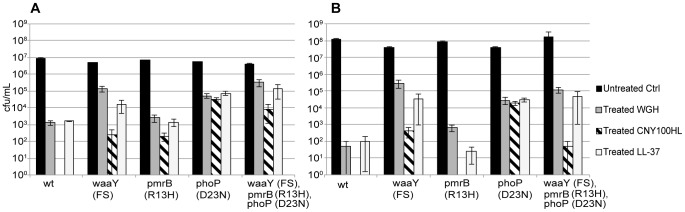
Survival of the reconstituted mutant strains. Cell viability for reconstituted strains (DA22427, DA23175, DA23307 and DA23899) and susceptible parental wild type strain (DA6192) at (A) 2 hours and (B) 4 hours of AMP-treatment. Black bars, untreated controls; dark gray bars, WGH treatment (10 mg/L); striped bars, CNY100HL treatment (5 mg/L); and light gray bars, LL-37 treatment (10 mg/L). The data ± SEM is derived from the time-kill assays in Figure 2.

Three selected mutations [*waaY* (del bp17FS), *pmrB* (R13H) and *phoP* (D23N)] were reconstituted in the wild type genetic background (resulting in strains DA22427, DA23175 and DA23307, respectively), and the resistance contribution of each mutation was determined in time-kill assays in the presence of AMPs (Fig. 2). Since the time-kill assay was not sensitive enough to reveal the subtle effect that *pmrB* (R13H) has on resistance we excluded it from Figure 2. The complete results are summarized in Figure 3, showing percent survival of the initial inoculum at 2 and 4 hours of AMP-treatment. The *waaY* mutation was originally identified in the WGH-passaged strain DA16875 (in addition, other *waaY* mutations were identified in LL-37 serially passaged strains DA16874 and DA17847). The *phoP* and *pmrB* mutations were originally found in the CNY100HL passaged strain DA17610 and in the WGH passaged strain DA16875 (in addition, another *pmrB* mutation was identified in LL-37 passaged strain DA16874). As can be seen in Figure 3b, the *waaY* mutation and the *phoP* mutation both contribute to resistance against all three peptides used in the study (between 2–4 logs higher survival than wild type at 4 h, respectively). The *pmrB* mutation conferred only a small increase in resistance for WGH in this assay (Figure 3). We further combined the mutations to examine if resistance could be increased further, but the double mutants behaved similarly to the *waaY* or *phoP* alone with the *pmrB* mutation having negligible effect (data not shown) and the triple mutant was slightly less resistant than, or had similar resistance levels as the *waaY* and *phoP* single mutants (Figures 2 and 3). The results are elaborated further below in the cross-resistance section.

### Fitness of AMP Resistant Mutants

Mutations, even ones that bring a conditional benefit such as antimicrobial resistance, typically come with a cost under non-selective conditions [32]. Fitness costs can be measured in single culture growth assays or in assays in which the mutant and wild type strains compete. In the growth rate analysis, the original resistant mutants all showed a reduction in fitness in all three media tested (Table 3). The WGH cycled mutant DA16875 had a similar fitness reduction in all three media: 12.6% in Mueller-Hinton (MH), 11% in refined LB and 12% in NaPB. The original CNY100HL resistant mutant DA17610 suffered the most fitness reduction, 17, 25 and 27% respectively, in the three different media. Both LL-37 cycled isolates had fitness costs of about 10% in NaPB and 4–6% in MH, but were differentially affected in refined LB; DA16874 had a fitness reduction of 12% while DA17847 only suffered a 2.7% reduction. The reconstituted strains were less affected in fitness (Table 3), which is expected given that they carry fewer mutations. The reconstituted *waaY* mutant only suffered from a 3.7% growth rate reduction in MH and a 1.1% reduction during competitions in NaPB. The *pmrB* single reconstituted mutant was not significantly affected in fitness, and this mutation may even be slightly advantageous in some media (a fitness increase of 4% and 0.8% in NaPB was determined from the growth rate assay and competition, respectively). The reconstituted *phoP* mutant had a fitness cost of ∼7 to 16% depending on the medium. The triple reconstituted mutant behaved similar to the *phoP* mutant alone. It should be noted that growth in NaPB is generally quite poor in Bioscreen plates (but not in competitions, see below) and therefore less reliable than growth rate determinations in refined LB and MH.

**Table 3:** Fitness of original and reconstituted mutants relative to wild type controls as determined by growth rate assays and competition experiments.

Strain	Fitness change relative to wild type			
	Growth rate assay			Competition assay
	Refined LB	MH	NaPB	NaPB
Wild type controls^a^	1	1	1	1
DA16875 (WGH resistant isolate 1)	0.89	0.87	0.88	ND
DA16874 (LL-37 resistant isolate 1)	0.88	0.94	0.91	ND
DA17847 (LL-37 resistant isolate 2)	0.97	0.96	0.90	ND
DA17610 (CNY100HL resistant isolate 1)	0.83	0.75	0.73	ND
DA22427 (*waaY* [FS] reconstituted)	ND	0.96	1.05	0.99
DA23175 (*pmrB* [R13H] reconstituted)	ND	0.99	1.04	1.01
DA23307 (*phoP* [D23N] reconstituted)	ND	0.93	0.84	0.84
DA23899 (*waaY*, *pmrB*, *phoP* reconstituted)	ND	0.83	0.91	0.84

a DA6079 was used as wild type control for the original mutants and the respective congenic strains were used as controls for reconstituted mutants.

ND, not determined.

Maximum standard error of the mean (SEM): Refined LB (SEM ±0.02), MH (SEM ±0.03) and NaPB (SEM ±0.05).

### Competition Assays in Presence of AMPs

**Figure 4:**
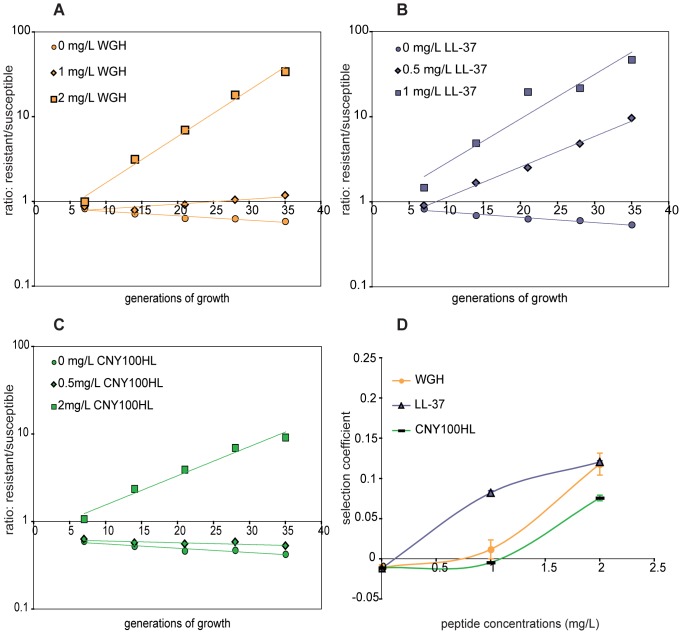
Competition experiments for the *waaY* (FS) mutant. Competitions between the reconstituted *waaY* frameshift mutant DA22427 (*cfp* tagged) and its congenic, susceptible counterpart DA22431 (*yfp* tagged). These experiments were performed at different concentrations of the three different peptides in this study, (A) WGHs (orange), (B) LL-37 (purple) and (C) CNY100HL (green). The curves represent the ratio of resistant (*waaY* mutant) to susceptible strain at different generations of growth. The data is the result of at least 2 separate competitions performed in duplicate for each peptide. (D) The selection coefficient ± SEM was deduced from the slopes in (A)-(C) and is plotted as a function of peptide concentration.

Although resistance mutations can confer a fitness cost and a disadvantage in comparison to the susceptible strain they have an advantage (i.e. resistance) in the presence of antimicrobials. If the benefit of resistance outweighs the burden of the fitness cost it is possible (at a certain concentration of AMPs) for the mutant to take over the population [24]. This can be analysed in competition assays in which the mutant and the congenic susceptible counterpart are mixed in equal proportions and allowed to compete in the absence and presence of different concentrations of the AMPs. This analysis was carried out for all reconstituted mutants and the results for the *waaY* mutation is shown in Figure 4. By plotting the ratio of resistant:susceptible bacteria as a function of time we obtain lines where the slope of each line represents the selection coefficient (s-value) (Fig. 4a–c). When the s values are plotted against AMP concentration we obtain the plot shown in fig. 4d, where the intercept on the X-axis indicates the concentration at which the fitness cost of the resistance is counter-balanced by the selection conferred by the AMP. This value is referred to as the minimal selective concentration (MSC) [24]. Importantly, th*e waaY* mutant always outcompeted the wild type in the presence of any of the three AMPs at concentrations around 1.0 mg/L (Fig. 4): for LL-37, WGH and CNY100HL these values were 0.1, 0.7 and 1 mg/L, respectively. For the *phoP* and *pmrB* single mutants variability between experiments was extensive, and we could therefore not obtain any reliable measurements of the MSC values even though the mutants occasionally outcompeted the wild type at low AMP concentrations (4–6 mg/L data not shown).

### Cross-resistance Studies

We next examined if any of the original or reconstituted AMP resistant strains exhibited cross-resistance to commonly used antibiotics or other AMPs as compared to the susceptible parental strain. Three different assays were used: (i) MIC determinations in liquid culture for AMPs (Table 4) and by E-tests for antibiotics (Table 5), (ii) time-kill assays at different AMP concentrations (Figs. 2 and 3) and (iii) competitions between susceptible congenic wild type and resistant mutants at different AMP concentrations (Fig. 4). MIC assays were used for the original mutant isolates and we observed no or modest cross-resistance or increased susceptibility to antibiotics (≤2fold increases/decreases, Table 5). Slightly higher changes (2- to 4-fold increases) were observed for the AMPs (Table 4).

**Table 4:** Cross-resistance to AMPs WGHs, LL-37 and CNY100HL as determined by microdilution MIC assays in NaPB.

Strains	MIC (mg/mL)		
	WGH	LL-37	CNY100HL
DA6192 (*S. typhimurium* wild type)	6.25	6.25	2.5
DA16875 (WGH resistant isolate 1)	25	6.25	2.5–5
DA17847 (LL-37 resistant isolate 2)	12.5–25	6.25	ND
DA16874 (LL-37 resistant isolate 1)	25	12.5–50	2.5–5
DA17610 (CNY100HL resistant isolate 1)	6.25–12.5	6.25–12.5	5
DA22427 (*waaY* [FS] reconstituted)	12.5	6.25	2.5
DA23175 (*pmrB* [R13H] reconstituted)	6.25–12.5	6.25	2.5
DA23307 (*phoP* [D23N] reconstituted)	6.25–12.5	6.25–12.5	5
DA23899 (*waaY, pmrB, phoP* reconstituted)	12.5–25	6.25	5

ND, not determined.

MIC determinations were performed on isolated clones obtained from the serially passaged populations (compare Table 2).

**Table 5:** Cross-resistance against different antibiotics as determined by Etests.

Strains	Antibiotic^a^ MIC (mg/L)						
	CR	FEP	MEM	CST	MEC	TGC	NAL
DA6192 (*S. typhimurium* wild type)	0.25	0.19	0.023	0.25	0.125	0.094	4
DA16875 (WGH resistant isolate 1)	0.25	0.25	0.023	0.19	0.125	0.064	1.5
DA17847 (LL-37 resistant isolate 2)	0.094	0.047	0.023	0.38	0.094	0.064	2
DA16874 (LL-37 resistant isolate 1)	0.19	0.047	0.032	0.19	0.19	0.064	2
DA17610 (CNY100HLresistant isolate 1)	0.19	0.064	0.032	0.25	0.125	0.064	4
DA22427 (*waaY* [FS] reconstituted)	0.25	0.125	0.023	0.25	0.064	0.064	3
DA23175 (*pmrB* [R13H] reconstituted)	0.25	0.125	0.032	0.25	0.094	0.094	4
DA23307 (*phoP* [D23N] reconstituted)	0.38	0.25	0.032	0.19	0.125	0.125	4
DA23899 (*waaY, pmrB, phoP* reconstituted)	0.25	0.19	0.047	0.5	0.125	0.094	2

a CR (cefpirome), FEP (cefepime), MEM (meropenem), CST (colistin), MEC (mecillinam),TGC (Tigecyclin), NAL (Nalidixic acid).

Similarly to the original mutants, the different reconstituted mutants conferred no or small cross-resistance/increased susceptibility against the tested antibiotics (≤2fold) but slightly higher cross-resistance against AMPs (2- to 4-fold) in the MIC assays (Tables 4–5). To further confirm the small differences observed in classical MIC determinations, we used time-kill assays and competition experiments. As can be seen in Figures 2 and 3, the *waaY* and *phoP* single mutants and the *waaY, phoP, pmrB* triple mutant were killed slower in the time kill assays as compared to the susceptible wild type strain, approximately 3 logs difference in killing at 4 hours. Similarly, in the competition assays the *waaY* mutant outcompeted the susceptible congenic control strain at low concentrations of WGH, LL-37 or CNY100HL (Fig. 4a–d), i.e. at concentrations where the control strain survives and grows. In combination, the time-kill and competition assays confirm that these mutations confer substantial cross-resistance to the three tested AMPs.

### Mutation Rate Determination

Bacterial strains with elevated mutation rates, so called mutators, are commonly found among antibiotic resistant isolates and tend to be enriched in certain environments [33]. To examine if mutators appeared during our selections, the mutation rates to rifampicin resistance of the different isolated mutants were determined and compared to that of the susceptible parental strain (Table 6). The WGH resistant isolate (DA16875), the CNY100HL resistant isolate (DA17610) and LL-37 resistant isolate 2 (DA17847) had mutation rates comparable to that of the wild type strain (2.7–6.9×10^−9^/cell/generation as compared to 4×10^−9^/cell/generation). In contrast, the mutation rate in LL-37 resistant isolate 1 (DA16874) was more than 600 times higher than that of the parental strain. This high mutation rate is reflected in the whole genome sequencing data, which revealed more than 80 mutations. Mutations in mismatch repair genes are often associated with elevated mutation rates. A 6 bp deletion in *mutL* was identified in this strain and by moving the *mutL* mutation into a wild type background we confirmed that this mutation is mainly responsible for the elevated mutation rate in strain DA16874 (Table 6). In addition, a second mutator was isolated from another LL-37 treated lineage (data not shown). That is, mutators were isolated from two out of six LL-37 treated lineages (lineages 2 and 5).

**Table 6:** Mutation rate to rifampicin resistance.

Strain	Mutation rate (per cell per generation)	Mutation rate relative to wild type
DA6079 (*S. typhimurium* wild type)	4.2×10^−9^	1
DA16874 (LL-37 resistant isolate1)	2.6×10^−6^	620
DA17847 (LL-37 resistant isolate 2)	2.7×10^−9^	0.64
DA16875 (WGH resistant isolate 1)	3.1×10^−9^	0.74
DA17610 (CNY100HL resistant isolate 1)	6.9×10^−9^	1.6
DA23040 (*mutL*(del aa 68–69), 4597041::Kan)	1.25×10^−6^	298
DA23042 (4597041::Kan)	4.1×10^−9^	0.98

## Discussion

Given that the initial serial passage experiment only ran for roughly 500 generations, it is alarming that several mutations connected with AMP resistance were obtained so readily. Previously it was surmised that microbial resistance development towards AMPs was improbable as the most common target of AMPs is the bacterial membrane, and bacteria would therefore be forced to rearrange their membranes dramatically which would be too “costly” in terms of fitness [3].

In this study we found and characterized mutations in *S. typhimurium* LT2, in the genes *waaY*, *phoP* and *pmrB*, that lead to AMP resistance and which are connected with lipopolysaccharide (LPS) modifications. The *waa* gene cluster is involved in LPS core biosynthesis and modification. The gene product of *waaY* adds a phosphate group to the heptose II residue of the LPS molecule [29], and mutations in *waaY* likely interrupt this process resulting in a reduced number of negative charges in the membrane and as a result, reduced interaction with positively charged AMPs. It has been shown before that mutations in other genes in the *waa* cluster result in a truncated LPS molecule and these mutations are typically referred to as having a “deep-rough” phenotype due to their rough appearance in colony formation [34]. This phenotype is also associated with increased susceptibility to certain cationic peptides (such as Polymyxin), hydrophobic antibiotics and loss of virulence [35]. It is well established that LPS biosynthesis genes work in a strictly stepwise manner to construct the LPS molecule [30]. However, the alteration in the *waaY* function appears to interfere little with the otherwise stepwise assembly of the rest of the LPS molecule since *waaY* (and *waaZ*) mutants do not have as serious phenotypes as many other mutants in the *waa*-pathway. For example, they do not exhibit the typical deep-rough phenotype and hyper-susceptibility to hydrophobic compounds [29].

The two-component signal transduction system *pmrAB* regulates several genes that have been connected to AMP resistance [36], [37]. For example, it also regulates phosphorylation of the heptose II residue via the *pmrG* gene. *PmrG* can remove a phosphate group to decrease the negative charge of the LPS [38]. In addition *pmrAB* controls expression of both the *pmrC* gene and the *pmrF* operon (*pmrEHIJKLM*). These genes directly control biosynthesis, transfer and addition of phosphoethanolamine and 4-amino-4-deoxy-L-arabinose (L-Ara4N) to the Lipid A portion of LPS, thus masking the bacterial membranes negatively charged residues [39]. Taken together, it is likely that both the *waaY* and *pmrB* mutations confer resistance by reducing or masking negative charges in the membrane [40], potentially decreasing the attraction of positively charged AMPs or making the membrane less accessible to the peptide. Our particular *pmrB* mutation (when reconstituted) only exhibited a small increase in resistance for WGH, which may be surprising given the many references in the literature of *pmrB* (or its signal transduction partner *pmrA*) being involved in AMP resistance [38], [41]. Since mutations in *pmrB* occurred in two out of the four cycled mutants that were whole genome sequenced, it is likely that the *pmrB* mutations were selected (and not accidental) under the specific growth regime used. To explain this discrepancy, we suggest that the *pmrB* mutations confer very small effects (as observed for WGH) and/or confer their effect only in combination with other mutations present in the original serially passaged strains. This phenomenon is referred to as epistasis. For example, the phenotype of *pmrB* (R13H) in this case could be dependent on the expression of one or more additional mutations found in the serially passaged strains (see Table S3). The *pmrB* mutation R13H was found in the same strain as the *waaY* frameshift mutation and might act in coordination to improve fitness without reducing resistance.

The *phoP* gene is the response regulator in the *phoPQ* two-component regulator system that influences expression of roughly 3% of the *Salmonella* genes and is essential for virulence in mice [42], [43]. It is directly responsible for controlling several genes that affect the cell wall outer monolayer consisting of primarily Lipid A, which is also the base of the LPS molecule [44], [45]. Lipid A modifications are described as being responsible for resistance to AMPs [46], [47]. For example, constitutive expression of *phoP* has been shown to modify the Lipid A through the addition of fatty acid chains- either 2-hydroxymyristate (mediated by *lpxO*) [48], [49], or palmitate (mediated by *pagP*) [46], [50]. These alterations are predicted to render the phospholipid bilayer less fluidic and more hydrophobic resulting in a outer membrane that is harder for AMPs to breach [46]. Furthermore, *phoP* regulates the *pmrAB* system and thus the resistance genes under control of these regulators [44]. Although we have not measured *phoP* activity/levels in the reconstituted strain (DA23307) we predict that this *phoP* mutation results in a constitutive on-state, due to the fact that it confers resistance to all three of the AMPs used in this study and *phoP* activated genes are known to be involved in resistance to AMPs [44], [49], [51].

Fitness measured as growth rate is a useful indicator of how probable it is for a mutant to survive in an environment wherein there are more fit organisms [32]. Most resistance mechanisms cause some reduction in fitness and if the fitness cost is sufficiently high, resistant bacteria may be out-competed by susceptible bacteria in the absence of the drugs. However, antibiotic resistant bacteria can overcome these fitness effects by acquiring additional mutations that compensate for the costs while retaining the original resistance mutation [32]. In addition, it has been demonstrated that resistant bacteria can have a growth advantage over the “more fit” wild type counterpart at antibiotic concentrations far below the MIC, and thus be maintained by very weak selection pressures [24].

We determined the fitness of a subset of the strains used in this study in the absence of AMPs. The original serially passaged AMP resistant strains suffered from fitness reductions between ∼3–30% depending on the media used. This result suggests that without selection pressure from AMPs, they would be outcompeted by their susceptible counterparts. The reconstituted mutants, especially the single mutants, generally have lower fitness costs (and the *pmrB* mutant may even have a slight advantage), making them better competitors. Based on our competition results, the *waaY* mutation is a likely candidate for persistence in the environment because of its low fitness cost (1.1%).

Furthermore, the low dosage of all three AMPs required to select for and maintain the *waaY* mutant in the population (∼0.1 mg/L–1.5 mg/L) is well within the concentration ranges of AMPs found in, for example, secretions near host epithelial cells [52], [53]. The alarming implication of these findings is that certain AMP resistant mutants (e.g. *waaY* and *phoP*) could both be selected *de novo* and then subsequently maintained by exposure to our own natural repertoire of defence molecules. Whether this is likely or not will largely depend on how well the *waaY* and *phoP* mutants grow and survive within a host. Since an intact LPS molecule (including the O-antigen) is important for virulence and survival inside hosts [54], [55], it is possible that the *waaY* mutants will not survive the host environment even in the presence of an AMP selective pressure. However, this will depend on the extent to which a *waaY* mutation influences LPS structure and membrane stability. Similarly, *phoP* deleted mutants in several bacterial species are avirulent in vivo [42], [56], [57]. However, as the *phoP* mutant isolated in our study is likely to be constitutive it is unclear how it would behave inside a host. Previous reports of a constitutive PhoP phenotype (caused by a *phoQ* mutation) demonstrate that *S. typhimurium* is attenuated for virulence, suggesting that a constitutive on-phenotype might also cause avirulence [51], [58]. Another potential complication that needs to be addressed is to what extent the fitness/virulence reduction conferred by these mutations can be compensated by second-site mutations without loss of resistance. Such compensatory evolution has been commonly observed for antibiotics and it is possible that similar processes might act on AMP resistant mutants [32]. An indication that these mutations would be able to survive and be selected *in vivo* comes from studies of clinical isolates of *P. aeruginosa*, where mutations in both the *pmrAB* and *phoPQ* systems have been identified to confer resistance to the peptide antibiotic polymyxin B (often a last available drug option for *Pseudomonas* infections) [59], [60].

A potential problem in pursuing the development and therapeutic use of AMPs as drugs is cross-resistance. Histones are highly conserved and although the histones used in this study are extracted from wheat germ, LL-37 is unaltered from the naturally occurring variant and CNY100HL is a peptide synthetically derived from the human C3a complement component leaving it with little homology to the original model sequence. Surprisingly, in spite of their different origins and structures we observe significant cross-resistance between these peptides for both the *phoP* and *waaY* mutants. The *phoP* mutant shows nearly equal resistance to all three peptides in the time kill assay. This pattern is also observed in the competition experiments with the *waaY* mutant in which, with concentrations well below the MICs of all three AMPs (i.e. where the wild type is able to survive and grow), it successfully outcompeted the susceptible parent strain. Cross-resistance was also tested against various antibiotics from different classes and although cross-resistance is seen against the AMPs used in this study, we found little (1–2 steps change in MIC) cross-resistance/susceptibility to antibiotics.

In this study we have used several types of assays (MIC, time-kill and competitions) to assess the level of resistance of the various mutants. It is notable that these assays vary extensively in sensitivity and that classical MIC assays (as determined by microdilution methods) are the least sensitive. For example, several reconstituted mutants showed no increase in MIC as measured by microdilution assays but in the other assays (time-kill and competitions) they were clearly less susceptible as compared to the susceptible parent strain. In light of these results, we suggest that MIC is less suitable for measuring bacterial resistance towards AMPs than time-kill or competition assays, especially if the mutants are not highly resistant.

Finally, although the need for new treatments is great it is important to try to carefully assess what would happen with regard to resistance development if we start to use AMPs as therapeutic agents in clinical settings. In the light of what we now know about the relative ease of resistance development to classical antibiotics, it is important to know more about resistance mechanisms and the resulting physiological effects on the bacteria for AMPs, to avoid repeating the same mistakes. A worst case scenario would be that resistance mechanisms are selected for that confers resistance to our normal repertoire of host defence peptides, ultimately rendering us more susceptible to infection [4]. Further research is needed on these AMPs and their potential for resistance development, so that the medical community proceeds carefully with regard to their potential clinical use.

## Supporting Information

Figure S1 **Growth of wild type** ***S. typhimurium*** **(DA6192) in refined LB in the presence of different antimicrobial peptides, as determined by OD measurements in a Bioscreen C analyzer. (a) LL-37, (b) CNY100HL, (c) Wheat germ histones.**
(DOCX)

Table S1 **MIC of LL-37, wheat germ histones and CNY100HL for wild type** ***S. typhimurium*** **(DA6192 and DA6079) in refined LB and 20** **mM Sodium-Phosphate buffer supplemented with 0.1% TSB.**
(DOCX)

Table S2 **Scar sequences (or Kan-insertions when relevant) in reconstituted strains constructed or used in this study.**
(DOCX)

Table S3 **Mutations identified in the whole genome sequencing data of LL-37 resistant isolate 1 (original mutant DA16874).** Genes examined in this study are marked in bold.
(DOCX)

Table S4 **Mutations identified in the whole genome sequencing data of LL-37 resistant isolate 2 (original mutant DA17847).** Genes examined in this study are marked in bold.
(DOCX)

Table S5 **Mutations identified in the whole genome sequencing data of Wheat germ histone resistant isolate 1 (original mutant DA16875).** Genes examined in this study are marked in bold.
(DOCX)

Table S6 **Mutations identified in the whole genome sequencing data of CNY100HL resistant isolate 1 (original mutant DA17610).** Genes examined in this study are marked in bold.
(DOCX)
