# Supplementary material for: Mechanisms and Fitness Costs of Resistance to Antimicrobial Peptides LL-37, CNY100HL and Wheat Germ Histones
Source: PLoS One. 2013 Jul 23;8(7):e68875. doi: 10.1371/journal.pone.0068875 (PMC3720879; doi:10.1371/journal.pone.0068875)
Supplement: Figure S1 — Growth of wild type S. typhimurium (DA6192) in refined LB in the presence of different antimicrobial peptides, as determined by OD measurements in a Bioscreen C analyzer. (a) LL-37, (b) CNY100HL, (c) Wheat germ histones. (DOCX) [file pone.0068875.s001.docx]

**Supplementary Figure S1.** Growth of wild type *S. typhimurium* (DA6192) in refined LB in the presence of different antimicrobial peptides, as determined by OD measurements in a Bioscreen C analyzer. (a) LL-37, (b) CNY100HL, (c) Wheat germ histones.
