# Supplementary material for: Mechanisms and Fitness Costs of Resistance to Antimicrobial Peptides LL-37, CNY100HL and Wheat Germ Histones
Source: PLoS One. 2013 Jul 23;8(7):e68875. doi: 10.1371/journal.pone.0068875 (PMC3720879; doi:10.1371/journal.pone.0068875)
Supplement: Table S1 — MIC of LL-37, wheat germ histones and CNY100HL for wild type S. typhimurium (DA6192 and DA6079) in refined LB and 20 mM Sodium-Phosphate buffer supplemented with 0.1% TSB. (DOCX) [file pone.0068875.s002.docx]

**Table S1.** MIC of LL-37, wheat germ histones and CNY100HL for wild type *S. typhimurium* (DA6192 and DA6079) in refined LB and 20 mM Sodium-Phosphate buffer supplemented with 0.1% TSB.

| **Peptide** | **MIC [mg/L]** | |
| --- | --- | --- |
|  | **Refined LB** | **NaPB+0.1% TSB** |
| CNY100HL | 5 | 2.5 |
| LL-37 | 12.5-50 | 6.25^a^ |
| WGH | 6.25-25 | 6.25 |

^a^Only measured for DA6079.
