# Supplementary material for: Mechanisms and Fitness Costs of Resistance to Antimicrobial Peptides LL-37, CNY100HL and Wheat Germ Histones
Source: PLoS One. 2013 Jul 23;8(7):e68875. doi: 10.1371/journal.pone.0068875 (PMC3720879; doi:10.1371/journal.pone.0068875)
Supplement: Table S2 — Scar sequences (or Kan-insertions when relevant) in reconstituted strains constructed or used in this study. (DOCX) [file pone.0068875.s003.docx]

**Table S2.** Scar sequences (or Kan-insertions when relevant) in reconstituted strains constructed or used in this study.

| **Scar designation** | **Sequence deleted^a^** | **Strains** |
| --- | --- | --- |
| *tdh*(scar) | 3902885-3903904  (in *tdh*) | DA22427, DA22431, DA23179, DA23299, DA23301, DA23305, DA23899, DA23902, DA23410, DA23418, DA23423, DA24148, DA24156, DA24161, DA24577, DA24593 |
|  |  |  |
| *adi*(scar) | 4539247-4540375  (in *adi*) | DA23175, DA23177, DA23179, DA23299, DA23899, DA23902, DA24144, DA24301, DA23414, DA23418, DA24152, DA24156, DA24577, DA24593 |
|  |  |  |
| 1327454(scar) | bp 1327454-1327514  (between STM1239 & *envF)* | DA23301, DA23305, DA23307, DA23309, DA23899, DA23902, DA24144, DA24301, DA23423, DA23426, DA24161, DA24164, DA24577, DA24593 |
|  |  |  |
| 4597041::Kan | bp 4597041-4597057  (between *glyY* and *yjeS*) | DA23040, DA23042 |

^a^Removal of the kanamycin cassette used for linear transformation by Flp recombinase results in an 85 nt scar sequence in place of the section of the genome that is deleted in the process.
