# Supplementary material for: Mechanisms and Fitness Costs of Resistance to Antimicrobial Peptides LL-37, CNY100HL and Wheat Germ Histones
Source: PLoS One. 2013 Jul 23;8(7):e68875. doi: 10.1371/journal.pone.0068875 (PMC3720879; doi:10.1371/journal.pone.0068875)
Supplement: Table S3 — Mutations identified in the whole genome sequencing data of LL-37 resistant isolate 1 (original mutant DA16874). Genes examined in this study are marked in bold. (DOCX) [file pone.0068875.s004.docx]

**Table S3.** Mutations identified in the whole genome sequencing data of LL-37 resistant isolate 1 (original mutant DA16874). Genes examined in this study are marked in bold.

SNV, single nucleotide change; Ins, insertion; Del, deletion; -, no amino acid change or not applicable.
